# Lifestyle and Socioeconomic Transition and Health Consequences of Breast Cancer in the East Asia Region, From 1990 to 2019

**DOI:** 10.3389/fnut.2022.817836

**Published:** 2022-04-11

**Authors:** Sumaira Mubarik, Jinhong Cao, Fang Wang, Syeda Rija Hussain, Qing Liu, Suqing Wang, Yan Liu, Chuanhua Yu

**Affiliations:** ^1^Department of Epidemiology and Biostatistics, School of Public Health, Wuhan University, Wuhan, China; ^2^Department of Biostatistics, School of Public Health, Xuzhou Medical University, Xuzhou, China; ^3^Department of Health Sciences, Rawalpindi Medical University, Rawalpindi, Pakistan; ^4^Department of Preventive Medicine, School of Public Health, Wuhan University, Wuhan, China; ^5^Global Health Institute, Wuhan University, Wuhan, China

**Keywords:** breast cancer, incidence, death, DALYs, risk factors, East Asia

## Abstract

**Background:**

Due to its higher prevalence and heterogeneity, female breast cancer (BC) is the widest disease throughout the world. We sought to assess the epidemiological and sociodemographic transitions of BC and to identify the potential risk factors attributed to burden of BC in East Asia.

**Methods:**

At the regional level of East Asia and at a national level of East Asian countries, we investigated the burden of the incidence of female BC, mortality, and disability-adjusted life years (DALYs) in 2019 and assessed the epidemiological, socioeconomic, and health-linked disparities in incidence of BC and mortality over a 30-year period. The changes in BC’s mortality and DALYs between 1990 and 2019, attributable to varying risk factors, were evaluated in different age groups.

**Results:**

In 2019, the incidence of and mortality from and DALYs of BC were estimated to be 382,321 (95% UI: 303,308–477,173) incidence cases [age-standardized rate (ASR) of 35.69 per 100,000; 28.32–44.54], 98,162 (79,216–120,112) deaths (ASR of 9.12; 7.36–11.13), and 3,024,987 (2,477, 984–3,659,370) DALYs with an ASR of 282.15 (230.81–341.19) in 2019. It was also observed that out of four most representative locations of East Asia, two (China and Japan) showed more than 60% increase in age-standardized incidence rate between 1990 and 2019. While only Japan females showed a significant rise of 15.3% (95% UI: 2.3–28) in ASR of death and 12.6% (95% UI: 0.5–26.9) in ASR of DALYs between 1990 and 2019. Inclusively, 88 and 81% variations were explained in the incidence of BC and death due to change in sociodemographic index (SDI) in 2019, in East Asia. The highest positive percent changes in death and DALYs between 1990 and 2019 were attributable to high body mass index (BMI), high fasting plasma glucose (FPG), and alcohol consumption in East Asia.

**Conclusion:**

The burden of death and disability from female BC is the result of multiple risk factors, mainly due to behavioral and metabolic risk factors. The increase of the incidence is related to the westernized lifestyle and diet habits and the improvement of screening and diagnosis techniques in the recent years, whereas the increase in DALYs is mainly attributed to high BMI, high FPG, alcohol use, and high diet in red meat.

## Introduction

Breast cancer (BC) has been considered as the disease of the western world in the past and is related to the epidemiological studies that were mainly conducted in the well-developed countries (1, 2). The Asia-Pacific region includes eastern, south-eastern Asia along with Oceania (forty-eight countries) and is the most population region of the world with almost 32% of the global female population (3). Due to the modification of lifestyle and reproductive patterns and the rapid growth of economy (4, 5), in the past two decades, the age-standardized incidence of BC in Asian countries has been raised at 4–8-folds than the global average (2). Although incidence of BC is still lower than the western countries, Asia’s contribution to the global burden of BC is aggravated rapidly because of its large population base (6).

Age-standardized incidence of BC varies from 27.0/100,000 in eastern Asia to 42.8/100,000 in western Asia (3). According to the estimates of Global Cancer Observatory 2018, BC is the top-most malignancy among women in China with age-standardized incidence of 36.1/100,000 and is highest incidence in east China (7, 8). Regardless of advance in medical science, Asian countries have also great variations in BC mortality due to the differences in environmental, socioeconomic, cultural lifestyle and health-care facility-related factors (9–11). Because most of the Asian countries are middle- and low-income countries, BC is still diagnosed in the advanced stages in these countries with inadequate resources as an early detection, diagnosis, and proper treatment cannot be proficiently endorsed. The survival rate for BC has been improved in developed countries (79%) as compared to developing countries (57%) possibly due to logistical and economical constraints (12, 13). In conclusion, BC illustrates as the major health issue with alarming situations in Asia (2, B14, B15).

Most of Asian countries are struggling to meet the basic living requirements, and it is difficult to maintain proper health-care systems. At present, only 20% of all Asian countries have their population-based cancer registries, of which only four countries covered the entire population (China, North Korea, Japan, and Singapore) (16). In the lack of representative information, updated usage of the available information can serve as a valuable tool to develop cancer-control policies. Another, exploring the regional- and national-level epidemiological and sociodemographic related transitions for the incidence of BC, mortality, and disability-adjusted life years (DALYs) will greatly assist in detecting the underlying causes, which leads to these variations, and may present indication based and regionally important information for public health policymakers. Majority of regional and national studies are based on the representation of current incidence of BC and mortality statistics (13, 17, 18) and do not shed light on the socioeconomic and lifestyle risk factor-related consequences. Moreover, research conducted on the global burden of BC over the last two decades varies from each other in terms of time interval used across the countries and commonly based on the short-term trends (19, 20). Therefore, we have planned not only to explore the temporal trends in the incidence, mortality, and DALYs of BC but also to shed light on the epidemiological and socioeconomic transitions over a 30-year period along with other disease-linked risk factors in the East Asian female population.

## Materials and Methods

### Study Population

Our study population comprised of the incidence of female BC, death, and DALYs, corresponding to the age group of 20–80+ years and age-standardized rate (ASR) of BC from 1990 to 2019 among the regional and national levels of East Asia. The epidemiological and socioeconomic transition-related index that is known as sociodemographic index (SDI), which is based on the national-level income per capita, average years of education among persons older than 15 years of age, and total fertility rate was used to assess the relationship between socioeconomic development and risk of BC. Data of the same outcome variables were also collected with varying risk factors that include metabolic risks, dietary risks, tobacco, and lifestyle-related risks by 5 year-interval of age group of 20–80+ years for 1990, 2000, 2010, and 2019.

### Data Sources

To assess the variations in BC over time, and related to multifactorial risk factors, we used the Global Burden of Diseases (GBDs) 2019 database. It provides a comprehensive assessment of 359 diseases and injuries, 282 causes of death, and 84 risk factors in 195 countries, 21 regions, and 7 super-regions (3–6). GBD provides the estimates of the incidence of BC, mortality, years lived with disability (YLD), years of life lost (YLL), and DALYs for 195 countries by sex and age group, with the most recent estimates applying to 2019.

The estimates of the incidence of BC were based on the individual cancer registries or integrated cancer registry databases. Mortality estimates were generated based on the data registries, which include consecutive annual data from national or subnational cancer registries, representing East Asian countries, for the longest period available (up to 2019). Registries details by location and cause have provided on GBD input source tool.^1^ Estimation framework of outcome variables is discussed in Supplementary Appendix Section 1. An overview of the analytic process is presented in Supplementary Figure 1.

### Attributable Risk Factors

The comparative risk assessment method has already been integrated into the GBD study (9) to measure the burden of multiple causes and impairments of 84 environmental, occupational, metabolic, and behavioral risk factors. Briefly, we chose two components to model the attributable burden of risk factors, which includes deaths and DALYs, following an assessment of the casual evidence in each risk-outcome pair.

### Statistical Analysis

We calculated percent change in the incidence of BC, mortality, and DALYs between 1990 and 2019 and presented the 2019 estimates with 95% uncertainty interval (UI) for East Asia regional and national levels. We also calculated this change for all ages and stratified by different age groups and risk factors. Geographical-wise assessment of BC variations was examined using map construction, stratified by age group and time. The relationship between country’s sociodemographic status (SDI) and BC (incidence and mortality) was assessed by visualizing both indexes correspondingly in the same years. Additionally, a regional polynomial regression curve fit was used to measure the evidence of this relationship by evaluating the coefficient of determination (R^2^). All analyses were performed using version 3.6.2 of the R packages and ArcGIS 10.2.

## Results

### Epidemiological Consequences of Breast Cancer at Regional and National Levels

**FIGURE 1:**
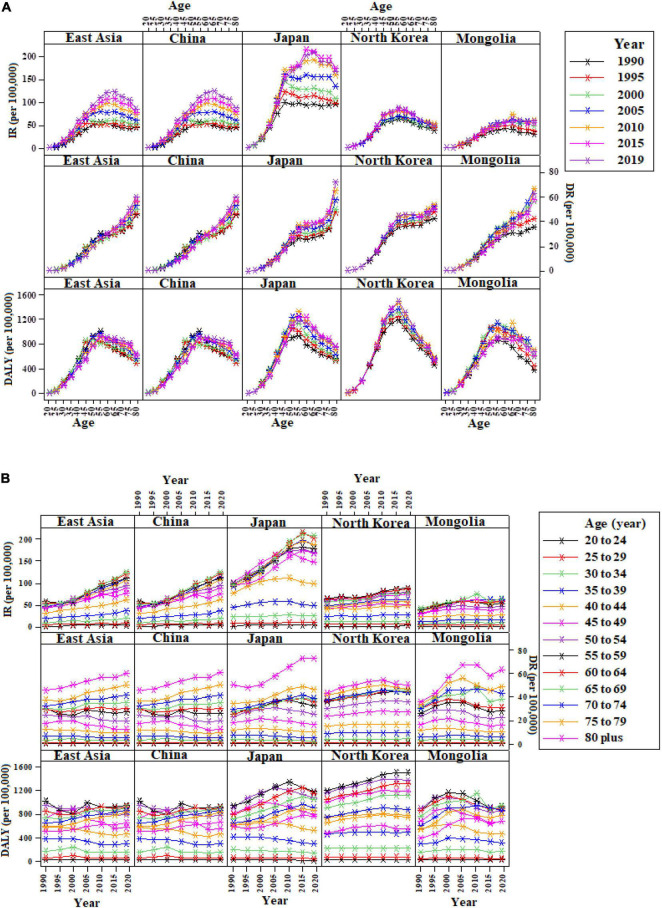
Incidence, death, and disability-adjusted life years (DALYs) trends by age and period for female breast cancer (BC) in East Asia. **(A)** Incidence, death, and DALYs trend by age in East Asia, 1990–2019; **(B)** incidence, death, and DALYs trend by period in East Asia, 20–80+ years. IR, Incidence rate; DR, Death rate.

Overall, in East Asia, BC among females accounted for 382,321 (95% UI: 303,308–477,173) incidence cases in 2019 (ASR of 35.7 [95% UI: 28.3–44.5]), which increased significantly by 107.1% (39.0–212.1) between 1990 and 2019. It was also observed that out of the four representative countries of East Asia, two (China and Japan) showed more than 60% increase in the age-standardized incidence rate between 1990 and 2019 (Table 1). The time-specific incidence trend over ages was almost in quadratic pattern, initially increases, and then starts to decline in each year from 1990 to 2019 in all East Asian regions under the study (Figure 1A). Meanwhile, higher age-specific incidence trend was observed in age group of 50–54 years onward during 1990–2019 (Figure 1B) for regions under the consideration.

**TABLE 1:** Incident cases, deaths, and disability-adjusted life years (DALYs) of breast cancer (BC) for females in 2019, and percentage change of age-standardized rates (ASRs) by regions between 1990 and 2019.

	Incidence (95% UI)			Deaths (95% UI)			DALYs (95% UI)		
	Incident cases, 2019	Age-standardized incidence (per 100 000 population), 2019	% Change in age-standardized incidence, 1990–2019	Death cases, 2019	Age-standardized mortality (per 100 000 population), 2019	% Change in age-standardized mortality, 1990–2019	DALYs cases, 2019	Age-standardized DALYs rate (per 100 000 population), 2019	% Change in age-standardized DALYs rate, 1990–2019
East Asia	382,321(303,308, 477,173)	35.7(28.3, 44.5)	107.1 (39.0, 212.1)	98,162 (79,216, 120,112)	9.1(7.4, 11.1)	−0.9 (−31.8, 44.9)	3,024,987 (2,477,984, 3,659,370)	282.2(230.8, 341.2)	−4.6 (−33.9, 38)
China	368,375 (290,086, 463,336)	35.6(28.1, 44.8)	108.7 (38.2, 219.5)	93,499 (74,511, 115,420)	9(7.2, 11.1)	−1.6 (−33.5, 45.9)	2,877,240 (2,323,688, 3,513,542)	278(224.3, 339.9)	−5.5 (−35.7, 39.6)
Japan	74,260 (59,292, 90,978)	60.5(48.3, 74.6)	67.7 (25.9, 119.8)	15,911 (13,374, 17,288)	10.1(9.2, 10.8)	15.3 (2.3, 28)	395,292 (355,850, 432,122)	338.1(314.5, 366.5)	12.6 (0.5, 26.9)
North Korea	4,658 (3,268, 6,447)	26.6(18.5, 37.1)	30.7 (−34.6, 159)	2,347 (1,726, 3,126)	13(9.5, 17.4)	18 (−36.9, 116.6)	74,530 (52,142, 102,531)	426.6(293, 595.1)	17.1 (−42.8, 131.1)
Mongolia	282 (200, 385)	17.9(12.9, 24)	46.1 (−18.8, 161)	140 (101, 190)	9.9(7.4, 13.2)	16.8 (−32.8, 105.1)	4,804 (3,397, 6,592)	292.7(210.5, 397.5)	7.2 (−41.4, 95.2)

Regarding death due to BC in females, it was documented 98,162 (95% UI: 79,216–120,112) deaths in 2019, with an ASR of 9.1 (95% UI: 7.4–11.1), which was identified as an insignificant decrease of 0.9% (95% UI: −31.8–44.9) in East Asia between 1990 and 2019. However, among the four East Asian countries, only China’s percent change in age-standardized mortality has a decreased trend of 1.6%, whereas Japan, North Korea, and Mongolia females showed the rise in trend, 15.3, 18, and 16.8%, respectively, between 1990 and 2019 (Table 1). While gradually, increasing mortality trend over ages and time was perceived in each year from 1990 to 2019 in all regions (Figure 1).

Additionally, the female BC’s DALYs in 2019 were 3,024,987 (95% UI: 2,477,984–3,659,370), ASR was 282.2 (95% UI: 230.8–341.2), and no significant changes in DALYs were observed between 1990 and 2019 in East Asia. At national level, only Japan indicated the significant rise of 12.6% (95% UI: 0.5–26.9) in ASRs of DALYs between 1990 and 2019 (Figure 1 and Table 1).

**FIGURE 2:**
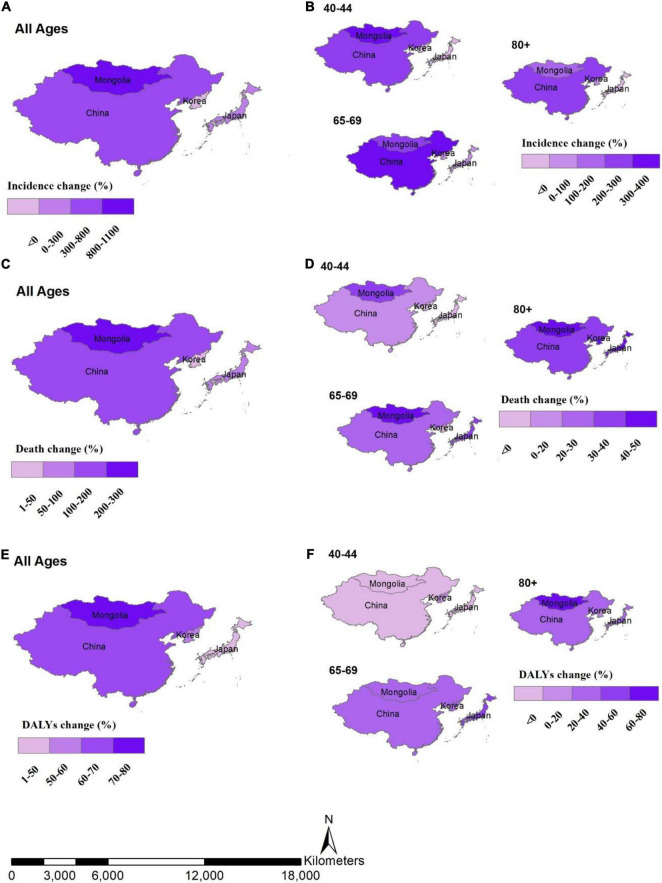
Percent change between 1990 and 2019, by location and age group, **(A)** incidence change in all ages by location, **(B)** incidence change by age groups, **(C)** death change in all ages by location, **(D)** death change by age groups, **(E)** DALYs change in all ages by location, **(F)** DALYs change by age groups.

The incidence of female BC and death rates among East Asian countries was also highly variable across the age groups (Figure 2). Incidence change (%) between 1990 and 2019 by age groups tended to be higher in 40–44 and 65–69 to 80+ years of age group in Mongolia and China, respectively. While the larger death percent change between 1990 and 2019 was observed in age group of 80+ years in all regions, the higher percent change in DALYs between 1990 and 2019 was in the age group of 65–69 years and 80+ years for all countries under the study (Figure 2).

### Sociodemographic Transition Linked Consequences of Breast Cancer at Regional and National Levels

**FIGURE 3:**
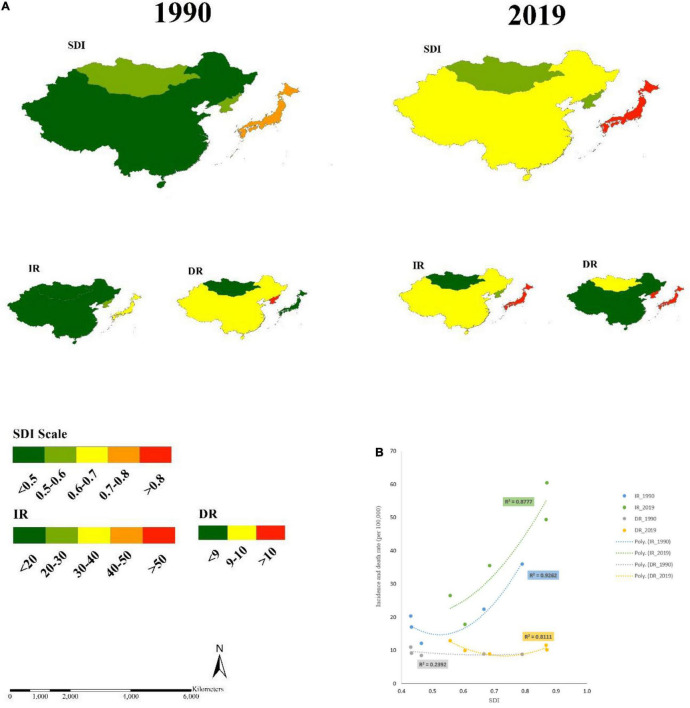
The relationship between SDI, and IR, and DR for female BC in East Asia, 1990 and 2019. **(A)** The link between SDI and DR for female BC, 1990 and 2019; **(B)** The strength of relationship between SDI and IR and DR of BC in East Asian female, 1990 and 2019; SDI, sociodemographic index; BC, breast cancer (rates per 100,000); IR, incidence rate; DR, death rate.

Overall, in East Asia, based on the four representative regions, 88 and 81% of the variations in the incidence of BC and death rates were explained by the changes in the SDI in 2019 (Figure 3B). There was also a sufficient link between SDI and BC among the countries. Figure 3A shows the relationship between the SDI and the incidence of BC and death rate at national level in 1990 and 2019. We observed that the countries had low SDI in 1990, and there were also the low incidence of BC, but high death rates. When we assessed this relationship for 2019, we noticed that the countries have increased SDI level in 2019, for example, China, and they also have increased the incidence rate of BC and reduced death rates, similar to that of 1990 in Japan. In summary, the incidence rate of BC was improved and death rate was reduced in the years of higher SDI measurements, for example, like in China and Japan.

### Risk Factor Linked Consequences of Breast Cancer at Regional and National Levels

**FIGURE 4:**
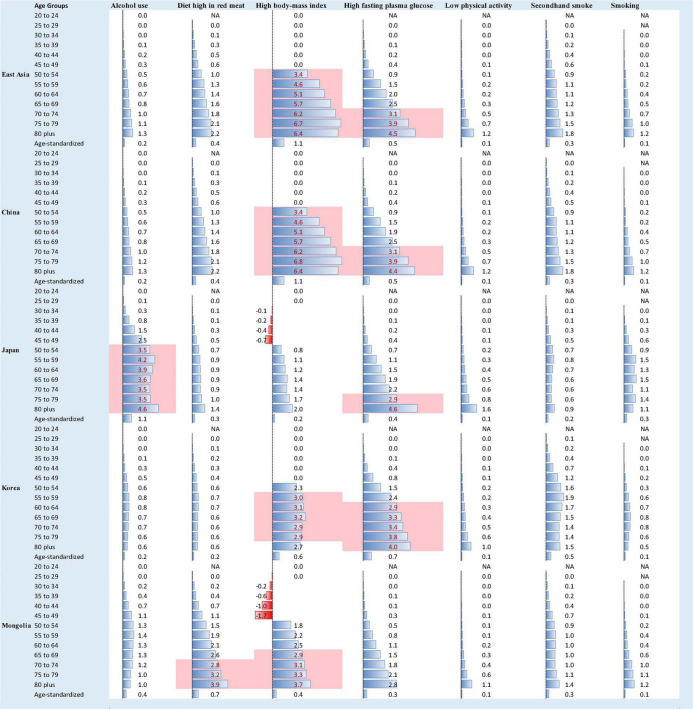
Female deaths (rate per 100,000) due to BC in 2019 stratified by age group 20 to 80+ years, age-standardized rate, and by risk factors in East Asia, the value with pink background fall in the rank of top 10%, NA = data are not available for those age groups and risk factors.

**FIGURE 5:**
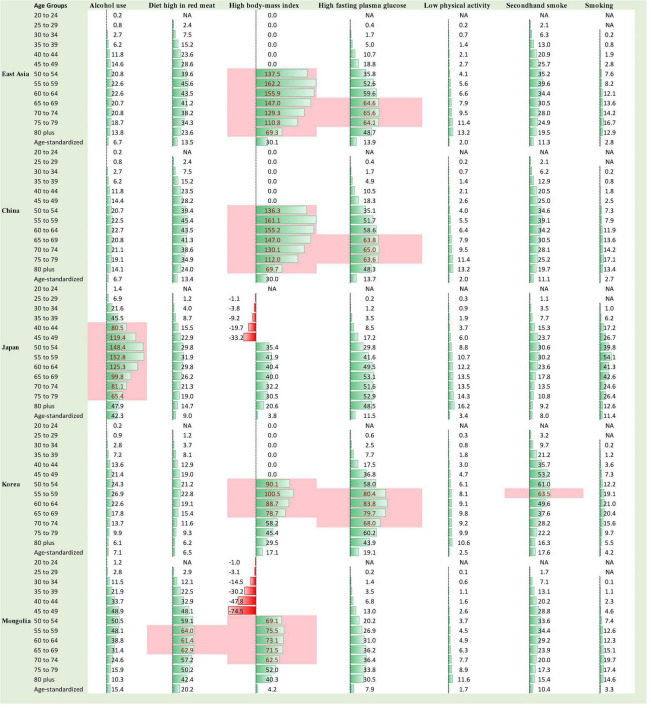
Female DALYs (rate per 100,000) due to BC in 2019 stratified by age group 20 to 80+ years, age-standardized rate, and by risk factors in East Asia, the value with pink background fall in the rank of top 10%, NA = data are not available for those age groups and risk factors.

Substantially different death and DALY patterns presented between different East Asian countries, which suggests that the risk factors for BC may differently influence the mortality and DALYs in those countries’ female population. The highest rate (per 100 k) of death and DALYs in 2019 was observed in these risk factors: high body mass index (BMI), high fasting plasma glucose (FPG), and high diet in red meat, particularly for age 50 onward. The top 10% of death and DALY rates in 2019 were related to high BMI and high FPG in all countries except Japan, where top 10% deaths were in risk factor of alcohol use (Figures 4, 5). The highest percent change in death and DALYs between 1990 and 2019 was attributable to high BMI, smoking, high FPG, and alcohol consumption at both regional and national levels. The pattern of risk factor related to death and DALYs in China was almost similar to that in East Asia as a whole. Mongolia showed the highest percent change in death and DALYs between 1990 and 2019, attributable to the alcohol use and high FPG, whereas for China, the highest percent change in risk attributable death and DALYs between 1990 and 2019 was observed in risk factor of high BMI (Supplementary Figure 2).

## Discussion

This study details the burden and trends of the incidence of BC, mortality, DALYs, and risk factors in East Asia from 1990 to 2019. It was estimated that there were huge increments in newly diagnosed cases, deaths, and DALYs of BC in East Asia within the last three decades. The age-standardized incidence of BC in East Asia has almost doubled, while the changes in age-standardized mortality and DALY rates were relatively stable. BC remains a major public health problem in East Asia, and it was heterogeneous compared to Europe and the United States.

### Potential Reasons for the Increased Burden of Breast Cancer in East Asia

Compared with North America and Europe, the prevalence of BC in East Asian populations (335.45 per 100 k in 2019) was relatively low, but its incidence was on the rise at a high speed, according to GBD2019. This could be attributed to the following:

#### Aging and Its Related Menopause

The risk of BC increases with age (21). In fact, the aging process, with what it carries with higher likelihood for genetic mutations, impaired physiological function, disturbed hormonal balance, etc., is the largest risk factor for BC. Despite the low risk of BC in postmenopausal than that of premenopausal women of the same age, with the natural age-related menopause, risk of BC increases by almost 3% for each year older at menopause (22). The rapid aging process in the East Asian countries could have attributed largely to the observed increased burden within the study period; 1990–2019. For example, the elderly population over the age of 65 years accounted for 27.6% of the total Japanese population in 2017, and it has been increasing (23). Meanwhile, Japan ranked first in terms of BC’s mortality, incidence, and age-standardized incidence rates among the studied East Asian countries. In Japan, the age-standardized death rate (ASDR) has been on the rise in the past 30 years, which indicates a high proportion of the elderly women. Our estimates showed that among Chinese women, the incidence of BC was low in women under the age of 30 years and then increased with the women’s age. The incidence peaks among women aged 60–69 years and the age group with the largest number of cases was the 50–59 years. The previous studies pointed out that the peak age of BC in Chinese women is about 50 years (24, 25). One important notice here is that the relatively unique birth pattern (the one-child policy) has reduced the fertility rate but may have increased the burden of BC; thus, China’s contribution to the global BC population also increases rapidly (36).

#### Screening Programs and Diagnostic or Therapeutic Techniques

High-income countries such as France and Canada started screening for BC in the late 1980s or early 1990s, and parts of Asia slowly followed (26). There is the growing evidence that over the past few decades, more BC cases have been detected due to the introduction of advanced new techniques for screening, which may have also influenced our study findings and contributed to the rising incidence in the Asian countries. Moreover, our data also show decreasing trend in age-standardized BC mortality and DALY rate from 2005 to 2015.

Japan officially recommended in 2004 that women should start mammograms at the age of 40 years, and once biannually after that (27). Clinical breast examinations, as well as mammograms, are widely available in public primary health-care institutions in Japan, which may also account for the high incidence of BC and declining trend of death and disability. However, there is no clear guideline for the termination age of screening, and elderly women do not pay enough attention to breast screening and self-examination, which also contributes to the high mortality rate of this group. Therefore, local government should also help Japanese women strengthen relevant health education and encourage women and the whole population to receive regular BC screenings. Chinese women have a 10-year earlier onset age of BC than that of European and American women (24, 25); therefore, the guidelines and specifications for the diagnosis and treatment of BC, issued by the Chinese Anti-Cancer Association, recommended 40 years as the starting age for BC screening for general population, and even before 40 years was recommended for the high-risk groups (28). Besides the early screening, the continuous development of current diagnostic and therapeutic technologies, such as X-ray, ultrasound (US), magnetic resonance imaging (MRI), and clinical physical examination for BC, and the clinical application of innovative drugs have provided a great progress in BC diagnosis (29).

The researchers have analyzed the relationship of BC mortality and disability with the availability of BC screening programs and comes up with the suggestion that both the variables have significant effect on international country-level health-care system. They have reported that BC screening either through mammography or digital mammography is strongly contributed to the decrease in mortality and disability of BC. Additionally, BC screening implementation only at national level causes significant reductions in BC mortality rates in comparison with no such screening programs. Low- and lower-middle-income countries are normally unable to provide population-based mammographic screening due to economic issues (30). Therefore, no proper data are available for such countries. But the evidence provided by the high-income countries is sufficient to convince the provision of clinical and self-examination facilities in the low- and lower-middle-income countries as cost-effective options (31–33). However, the knowledge related to efficacy of such screening attempts is limited, and this is immediately required to expand the influence of screening practices where it is needed the most.

#### Socioeconomic Transition

This study revealed that 88 and 81% of the total variations in incidence of BC and death in 2019 in East Asia were explained by changes in SDI. The research in China has shown that women in low socioeconomic status areas were more likely to be diagnosed with BC at an advanced stage than women in areas with high economic levels (34). According to the WHO, a large number of deaths due to BC are still occurring in low- and middle-income countries, where there is a lack of awareness about an early detection of the disease and possible barriers to health services, which leads to the significant number of women with BC who are not diagnosed until the disease is advanced. Such scenarios can, of course, be greatly improved or reversed if proper public health planning is established.

#### Risk of Breast Cancer Attributable to Modified Risk Factors

Not only the above-mentioned factors were logically contributed to the burden of BC in East Asia, but also with the population growth, the extension of life expectancy, and the aggravation of aging in Asia, the acceleration of global economic integration, which has brought about major changes in production and lifestyle with intensification of urbanization and the popularization of western lifestyle, has contributed to the rising burden of BC (35).

Our study findings revealed that the risk factors of BC’s DALYs in East Asia have changed during the 30 years from 1990 to 2019. Major risk factors have changed from behavioral to metabolic risk factors, such as high FPG, high BMI, and high diet in red meat, which were the top-three risk factors, followed by second-hand smoke, alcohol use, smoking, and low PA.

#### Fasting Plasma Glucose

We explored the risk factors in different East Asian countries and found that in China, Japan, Mongolia, and Democratic People’s Republic of Korea (DPRK), high FPG ranked among the top-three attributions of risk factors for BC’s DALYs. FPG is a high-risk factor for BC, especially around menopause (26). Diabetes is a risk factor for BC, and it is also an important factor that affects the worse prognosis of BC (26). FPG can also increase the risk of postoperative recurrence of BC, and high blood glucose levels can produce free radicals that can induce damage to DNA repair enzymes, which hinders the DNA repair and causes cancer (36). In addition, the prognosis was worse than that of patients with non-diabetes (37).

#### Body Mass Index

Also, the studies have shown that the incidence of BC increases with BMI (27, 38). Obesity was also an important risk factor for BC recurrence and death (34). The enhanced aromatase activity and high estrogen levels in patients with obesity can promote the excessive proliferation of breast tissue and inhibit cell apoptosis, which leads to the occurrence and progression of BC (24, 39).

#### Red Meat

A meta-analysis of prospective studies that investigated the association between meat intake and risk of BC has shown that the pooled RR (95% CI) of BC for the highest vs. lowest categories of red meet intake was 1.10 (1.02–1.16), and each additional 120 g of red meat per day was associated with 11% extra risk of BC (25). The potential biological mechanisms include the heme iron and non-heme iron prooxidant activity and also the carcinogenic by-product compounds that are produced in the process of high-temperature cooking of red meat. The consumption of red meat has been increased marvelously in the East Asian countries in the last few decades and is expected to be one of the regional areas with the high consumption of meat by 2030 (40).

#### Other Factors

Globally, alcohol consumption has increased over the past 30 years, especially in low- and middle-income countries, at a higher growth rate than in high-income countries (41). A pooled analysis of 20 prospective cohort studies suggested that alcohol consumption is positively associated with the overall risk of BC and BC subtypes, with stronger associations in postmenopausal women than in premenopausal women (42). In Japan, the BC’s DALYs caused by alcohol use were much higher than that in other countries, with an increase of 23.97% in the past 30 years from 1990 to 2019. The DALYs caused by the alcohol use in 2019 were 4 times that of Mongolia and 7 times that of DPRK. In Japanese postmenopausal women, heavy alcohol consumption was significantly associated with an increased risk of BC (43).

Smoking and second-hand smoke were causally related to many types of cancer that include BC. A study showed a consistent dose–response association between the number of years of smoking before the first childbirth (HR, 1.60; 95% CI, 1.42–1.80) and the risk of developing BC in short-term and long-term smokers (44). The proportion of East Asian females who smoke or exposed to second-hand smoking was on the rise during the study period (45).

A meta-analysis of 38 cohort studies showed that physically inactive women were at a greater risk of BC than physically active women (42). Mechanisms linked this increased risk with obesity in physically inactive women which was associated with risk of BC, recurrence, and mortality.

### Limitations

Our research results were estimated by integrating data from multiple sources in the GBD network and resources of multiple collaborators, and using a constantly updated complex modeling process, however, there are some limitations in our study. First, we described GBD’s estimates of the burden of BC, although based on the multi-source data currently available, but may not be accurate due to its wide range of estimates. Second, we have described only the overall trends of each country, but did not describe the sub-administrative regions of each country further. Although the results may vary to some extent from the original scenario, but even then, it is best to give at least a perspective of the diagnosis of the disease and its dangerous effect in the form of mortality that arouses the government and local bodies, which should maintain accurate records and ultimate requirements for disease diagnosis and management. Therefore, GBD data are suitable for comparing trends between different countries at present, despite the uncertainty issues related to measurement errors, missing data, and systematic bias of the countries’ prior estimates of the risk factors and BC pooled in the GBD.

## Conclusion

Overall, this study estimated the trends of BC in four Asian countries (China, Japan, North Korea, and Mongolia) over 30 years from 1990 to 2019, which shows that the burden of BC has been increasing and remains a significant public health problem in East Asia. The burden of death and disability from BC is the result of multiple risk factors, mainly behavioral and metabolic risk factors. The increase of the incidence was related to the westernized lifestyle and diet habits and the improvement of screening and diagnosis techniques in the recent years besides the aging process of the region, while the increase in BC’s DALYs was mainly attributed to high BMI, high FPG, alcohol use, and high diet in red meat. Therefore, East Asians need to operate, more efficiently, the BC screening programs, take effective interventions for major risk factors, reduce unhealthy eating habits, control the use of alcohol and tobacco, control weight gain, and increase physical activity.

## Data Availability Statement

Publicly available datasets were analyzed in this study. This data can be found here: Institute for Health Metrics and Evaluation (IHME): http://ghdx.healthdata.org/gbd-results-tool.

## Author Contributions

SM: conceptualization, data curation, formal analysis, methodology, software, validation, visualization, writing-original draft, and writing-review and editing. JC: conceptualization, investigation, methodology, and visualization. FW: investigation and writing-review and editing. SRH: visualization and writing-review and editing. QL and YL: validation, visualization, and data curation. SW: data curation, methodology, visualization, and investigation. CY: funding acquisition, investigation, project administration, resources, supervision, and validation. All authors contributed to the article and approved the submitted version.

## Conflict of Interest

The authors declare that the research was conducted in the absence of any commercial or financial relationships that could be construed as a potential conflict of interest.

## Publisher’s Note

All claims expressed in this article are solely those of the authors and do not necessarily represent those of their affiliated organizations, or those of the publisher, the editors and the reviewers. Any product that may be evaluated in this article, or claim that may be made by its manufacturer, is not guaranteed or endorsed by the publisher.

## Footnotes

1
https://ghdx.healthdata.org/gbd-2019/data-input-sources
