# Supplementary material for: Lifestyle and Socioeconomic Transition and Health Consequences of Breast Cancer in the East Asia Region, From 1990 to 2019
Source: Front Nutr. 2022 Apr 11;9:817836. doi: 10.3389/fnut.2022.817836 (PMC9036067; doi:10.3389/fnut.2022.817836)
Supplement: Supplementary file 1 [file Data_Sheet_1.pdf]

Supplemental Information:

Lifestyle and socioeconomic transition and health consequences of breast cancer in the East Asia region, from 1990 to 2019

Contents

Estimation framework of outcome variables…………………………………………….....................................2

Appendix Table 2: CODEm covariates used, level of covariate, and expected direction of covariate for breast cancer by age...5

Supplemental Figure S1: Analytical process overview…………………………………………………..….8

Supplemental Figure S2. Percent change in female BC deaths and DALYs between 1990 and 2019, stratified by age groups (20 to 80+yrs), age standardized, and by different risk factors in East Asia…………………………….9

References…………………………………………………………………………………………….....10

Section 1. Estimation framework of outcome variables

Breast cancer incidence estimates were based on individual cancer registries or integrated cancer registry databases. In order to find evidence of breast cancer death due to the attributable risk factors (alcohol consumption, high body mass index, high fasting plasma glucose, diet high in red meat, low physical activity, smoking and second-hand smoke), a systematic literary search in Pub Med took place. The proportions of breast cancer cases caused by different risk factors were calculated for each study parameter included. Four independent DisMod-MR2.1 inputs were applied to the proportion of data obtained from the systematic literature review.[1] Breast cancer mortality data from vital registration systems and mortality projections were used as input data into CODEm (Death Ensemble Model)[2]. The CODEm estimates mortality on the basis of available data and covariates, including education, smoking, SDI, lagging distribution income, and alcohol consumption (Appendix table 1). Using the CodCorrect procedure, the single cause estimates have been modified to fit all-cause mortality calculated separately.[3,4] Years lived with disability (YLDs) were determined by multiplying the prevalence of each sequela by its weight of disability and adding the clinical morbidity associated with a breast cancer diagnosis. Years of life lost (YLLs) caused by breast cancer have been estimated using global standard life expectancy and number of deaths by age.[3] DALYs for breast cancer have been computed as the sum of YLDs and YLLs.

This study also included the association between sociodemographic index (SDI) and breast cancer incidence, death and DALYs among East Asian regions. SDI is a composite indicator of lag-dependent income per capita, average years of schooling for the population older than 15 years of age, and total fertility rate under the age of 25. This index ranges from 0 (less developed) to 1 (most developed). Additional details for calculation of SDI have been presented previously.[5]

1.1 DisModMR-2.1 likelihood estimation

According to GBD-2019 study[6], the default log-Gaussian equation for data likelihood is

$$-log[p(y)i|\Phi] = log(\sqrt{2\pi}) + log(\delta_j + s_j) + \frac{1}{2}\left(\frac{log(a_j + \eta_j) - log(m_j + \eta_j)}{\delta_j + s_j}\right)^2$$

Where, y_j is a measurement value (i.e., data point), Φ denotes all model random variables, η_j is the offset value (prevalence, incidence, remission, excess mortality rate, with-condition mortality rate, cause-specific mortality rate, relative risk, or standardized mortality ratio) and a_j is the adjusted measurement for data point j, defined by

$$a_j = e^{(-u_j - c_j)} y_j$$

Where, u_j is the total area effect (i.e., the sum of the random effects at three levels of the cascade: super-region, region, and country) and c_j is the total covariate effect (i.e., the mean combined fixed effects for sex, study-level, and country-level covariates), defined by

$$c_j = \sum_{k=0}^{K[I(j)]-1} \beta_{I(j),k} \hat{X}_{k,j}$$

with standard deviation (SD), $s_j = \sum_{l=0}^{L[I(j)]-1} \zeta_{I(j),l} \hat{Z}_{k,j}$

Where, k denotes the mean value of each data point in relation to a covariate (x-covariate), $I(j)$ denotes a data point for a particular integrand, j, while, $\beta_{I(j),k}$ is the multiplier of the kth x-covariate for the ith integrand, $\widehat{X}_{k,j}$ is the covariate value corresponding to the data point j for covariate k, l denotes the SD of each data point in relation to a covariate (also called z-covariate), $\zeta_{Ij,k}$ is the multiplier of the lth z-covariate for the ith integrand, δ_j is the SD for adjusted measurement j, defined by

$$\delta_j = log[y_j + e^{(-u_j - c_j)}\eta_j + c_j] - log[y_j + e^{(-u_j - c_j)}\eta_j]$$

Where m_j denotes the model for the jth measurement, not counting effects of measurement noise and defined by

$$m_j = \frac{1}{B(j) - A(j)} \int_{A(j)}^{B(j)} I_j\,(a)da$$

Where, Aj is the lower bound of the age range for a data point j, Bj is the upper bound of the age range for a data point j, Ij denotes the function of age corresponding to the integrand for data point j.

1.2 Data transformation

Original data were standardized in aspect of format, categorization and registry names. Then cancer registry incidence data and mortality data are mapped separately into GBD causes. The cancer registry data were then standardized to the GBD age groups. Age-specific incidence rates were generated using all datasets with available microdata, and age-specific mortality rates were generated from the CoD data through a age-sex splitting method[6].

1.3 Calculation of age-specific mortality rates

The age-sex splitting was used to generate the age-specific mortality rates, which based on the following formula:

$$D_a = R_a N_a (\frac{D_a^{a+x}}{\sum_a^{a+x}(R_a N_a)})$$

Where, D_a = the number of deaths from a cause in age group a, R_a = global cause-specific mortality rate of age group a, N_a = the country-year-sex-specific population in age group a, D_a^{a+x} = the number of deaths in the age group a to $a + x$ with the assumption of invariant relative risk of death by age with respect to a reference age group, this equation can be used, along with population distribution by age, to split an aggregate number of deaths for the age groups a to $a + x$ into specific deaths for each age group within the aggregated interval.

$$D_{as} = R_{as} N_{as} (\frac{D_{as}^{a+x,s}}{\sum_a^{a+x}(R_{as} N_{as})})$$

Where, D_{as} = the number of deaths from a cause in age group a, sex s, R_{as} = global cause-specific mortality rate of age group a, sex s, N_{as} = the country-year-sex-specific population in age group a for sex $s, D_{as}^{a+x,s}$ = the number of deaths in the age group a to $a + x$ for sex s, Age-specific rates for a given registry population was then applied to generate age specific proportions, the expected number of cases/deaths for that registry by age was then produced. The expected number of cases/deaths for each sex, age, and cancer were then normalized to 1, creating final, age-specific proportions, and was then applied to the total number of cases/deaths by sex and cancer to get

the age-specific number of cases/deaths. Age-sex splitting was used for cases when cancer registry does not provide death number and incidence number by specific sex or age groups, and causes were disaggregated if the registries report provide causes as aggregated causes. The unspecified codes (garbage codes) are redistributed using the cause of death database, which has been explained elsewhere.[6]

Appendix Table 1: CODEm covariates used, level of covariate, and expected direction of covariate for breast cancer by age

Cause	Sex	Age Start	Age End	Model Type	Direction	Level	Covariate Name
Breast cancer	Female	15-19 years	95+ years	Data Rich	1	1	Mean BMI
Breast cancer	Female	15-19 years	95+ years	Data Rich	1	1	Log-transformed SEV scalar: Breast C
Breast cancer	Female	15-19 years	95+ years	Data Rich	1	1	Alcohol (liters per capita)
Breast cancer	Female	15-19 years	95+ years	Data Rich	-1	2	Healthcare access and quality index
Breast cancer	Female	15-19 years	95+ years	Data Rich	-1	2	vegetables adjusted(g)
Breast cancer	Female	15-19 years	95+ years	Data Rich	-1	2	Total Fertility Rate
Breast cancer	Female	15-19 years	95+ years	Data Rich	-1	2	Age-Specific Fertility Rate
Breast cancer	Female	15-19 years	95+ years	Data Rich	-1	2	fruits adjusted(g)
Breast cancer	Female	15-19 years	95+ years	Data Rich	1	2	Secondhand smoke
Breast cancer	Female	15-19 years	95+ years	Data Rich	1	2	Smoking Prevalence
Breast cancer	Female	15-19 years	95+ years	Data Rich	1	2	Cumulative Cigarettes (5 Years)
Breast cancer	Female	15-19 years	95+ years	Data Rich	1	2	Cumulative Cigarettes (10 Years)
Breast cancer	Female	15-19 years	95+ years	Data Rich	1	2	Cumulative Cigarettes (15 Years)
Breast cancer	Female	15-19 years	95+ years	Data Rich	1	2	Cumulative Cigarettes (20 Years)

Breast cancer	Female	15-19 years	95+ years	Data Rich	1	2	Diabetes Fasting Plasma Glucose (mmol/L)
Breast cancer	Female	15-19 years	95+ years	Data Rich	-1	3	Education (years per capita)
Breast cancer	Female	15-19 years	95+ years	Data Rich	0	3	Socio-demographic Index
Breast cancer	Female	15-19 years	95+ years	Data Rich	0	3	LDI (I$ per capita)
Breast cancer	Female	15-19 years	95+ years	Global	1	1	Mean BMI
Breast cancer	Female	15-19 years	95+ years	Global	1	1	Log-transformed SEV scalar: Breast C
Breast cancer	Female	15-19 years	95+ years	Global	1	1	Alcohol (liters per capita)
Breast cancer	Female	15-19 years	95+ years	Global	-1	2	Healthcare access and quality
							index
Breast cancer	Female	15-19 years	95+ years	Global	-1	2	vegetables adjusted(g)
Breast cancer	Female	15-19 years	95+ years	Global	-1	2	Total Fertility Rate
Breast cancer	Female	15-19 years	95+ years	Global	-1	2	Age-Specific Fertility Rate
Breast cancer	Female	15-19 years	95+ years	Global	-1	2	fruits adjusted(g)
Breast cancer	Female	15-19 years	95+ years	Global	1	2	Secondhand smoke
Breast cancer	Female	15-19 years	95+ years	Global	1	2	Smoking Prevalence
Breast cancer	Female	15-19 years	95+ years	Global	1	2	Cumulative Cigarettes (5 Years)

Breast cancer	Female	15-19 years	95+ years	Global	1	2	Cumulative Cigarettes (10 Years)
Breast cancer	Female	15-19 years	95+ years	Global	1	2	Cumulative Cigarettes (15 Years)
Breast cancer	Female	15-19 years	95+ years	Global	1	2	Cumulative Cigarettes (20 Years)
Breast cancer	Female	15-19 years	95+ years	Global	1	2	Diabetes Fasting Plasma Glucose (mmol/L)
Breast cancer	Female	15-19 years	95+ years	Global	-1	3	Education (years per capita)
Breast cancer	Female	15-19 years	95+ years	Global	0	3	Socio-demographic Index
Breast cancer	Female	15-19 years	95+ years	Global	0	3	LDI (I$ per capita)

Supplemental Figure S1: Analytical process overview

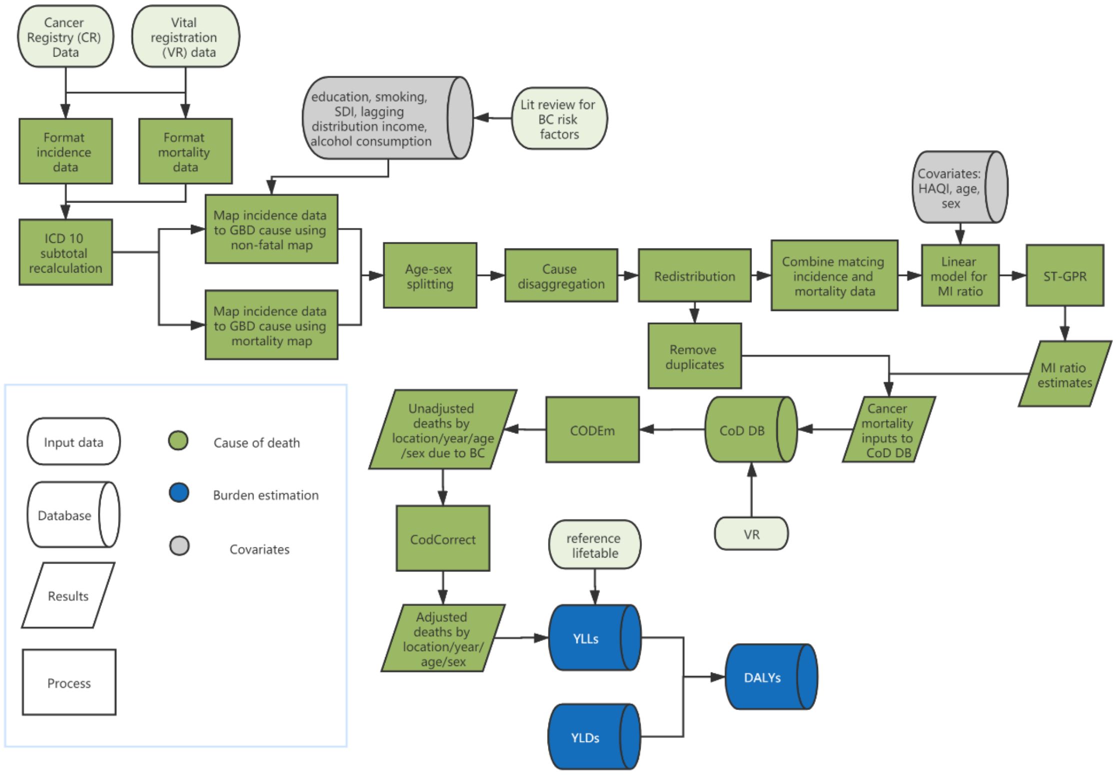

Supplemental Figure S2. Percent change in female BC deaths and DALYs between 1990 and 2019, stratified by age groups (20 to 80+yrs), age standardized, and by different risk factors in East Asia; Gray colour (NA), indicate data is not available for those age groups and risk factors, Korea= Democratic People's Republic of Korea (DPRK), BC=Breast cancer (rate per 100k), DALYs= Disability adjusted life years; + sign indicate positive change; - sign indicate negative change

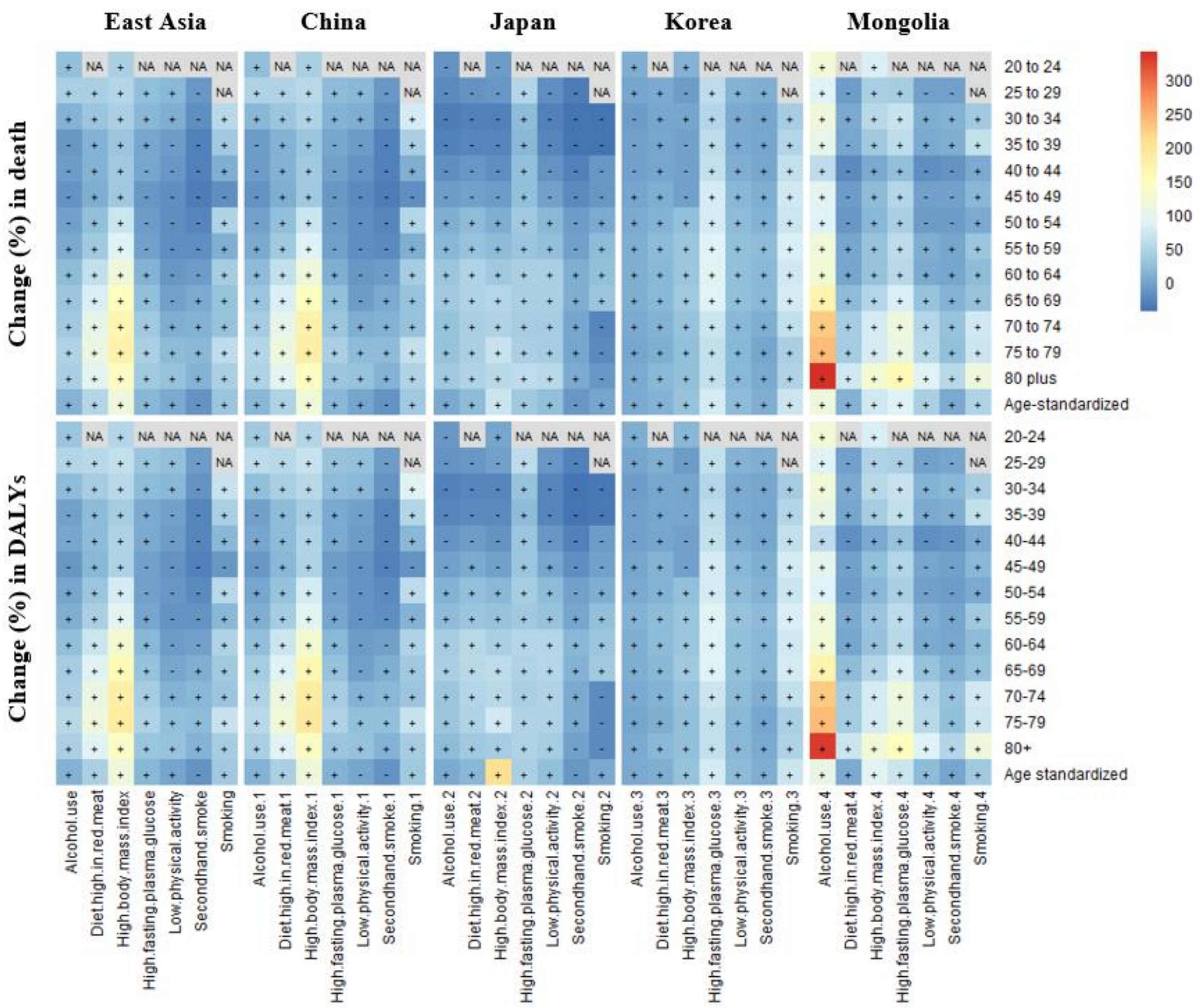

References

1. Vos T, Abajobir AA, Abate KH, et al. Global, regional, and national incidence, prevalence, and years lived with disability for 328 diseases and injuries for 195 countries, 1990–2016: a systematic analysis for the Global Burden of Disease Study 2016. *The Lancet* 2017; **390**(10100): 1211-59.
2. Foreman KJ, Lozano R, Lopez AD, Murray CJ. Modeling causes of death: an integrated approach using CODEm. *Population health metrics* 2012; **10**(1): 1.
3. Wang H, Abajobir AA, Abate KH, et al. Global, regional, and national under-5 mortality, adult mortality, age-specific mortality, and life expectancy, 1970–2016: a systematic analysis for the Global Burden of Disease Study 2016. *The Lancet* 2017; **390**(10100): 1084-150.
4. Zhou L, Deng Y, Li N, et al. Global, regional, and national burden of hodgkin lymphoma from 1990 to 2017: estimates from the 2017 Global Burden of Disease Study. *Journal of hematology & oncology* 2019; **12**(1): 107.
5. James SL, Abate D, Abate KH, et al. Global, regional, and national incidence, prevalence, and years lived with disability for 354 diseases and injuries for 195 countries and territories, 1990–2017: a systematic analysis for the Global Burden of Disease Study 2017. *The Lancet* 2018; **392**(10159): 1789-858.
6. Vos T, Lim SS, Abbafati C, et al. Global burden of 369 diseases and injuries in 204 countries and territories, 1990–2019: a systematic analysis for the Global Burden of Disease Study 2019. *The Lancet* 2020; **396**(10258): 1204-22.
